# New insight into the granule formation in the reactor for enhanced biological phosphorus removal

**DOI:** 10.3389/fmicb.2023.1297694

**Published:** 2023-12-14

**Authors:** Anna Pelevina, Evgeny Gruzdev, Yulia Berestovskaya, Alexander Dorofeev, Yury Nikolaev, Anna Kallistova, Alexey Beletsky, Nikolai Ravin, Nikolai Pimenov, Andrey Mardanov

**Affiliations:** ^1^Winogradsky Institute of Microbiology, Federal Research Center of Biotechnology, Russian Academy of Sciences, Moscow, Russia; ^2^K.G. Skryabin Institute of Bioengineering, Federal Research Center of Biotechnology, Russian Academy of Sciences, Moscow, Russia

**Keywords:** phosphorus removal, phosphate-accumulating bacteria (PAO), *Candidatus* Accumulibacter, formation of granule-like aggregates, EBPR

## Abstract

While granulated activated sludge exhibits high productivity, the processes of granule formation are incompletely studied. The processes of granule formation and succession of communities were investigated in a laboratory sequencing batch reactor (SBR) under conditions for enhanced biological phosphorus removal (EBPR) using microbiological and molecular techniques. Active consumption of acetate, primarily by the phosphate-accumulating organisms (PAO), commenced at day 150 of cultivation. This was indicated by the high ratio of molar P-released/acetate uptake (0.73–0.77 P-mol/C-mol), characteristic of PAO. During this period, two types of granule-like aggregates formed spontaneously out of the activated sludge flocs. The aggregates differed in morphology and microbial taxonomic composition. While both aggregate types contained phosphorus-enriched bacterial cells, PAO prevailed in those of morphotype I, and glycogen-accumulating organisms (GAOs) were predominant in the aggregates of morphotype II. After 250 days, the elimination of the morphotype II aggregates from the reactor was observed. The subsequent selection of the community was associated with the development of the morphotype I aggregates, in which the relative abundance of PAO increased significantly, resulting in higher efficiency of phosphorus removal. Metagenomic analysis revealed a predominance of the organisms closely related to *Candidatus* Accumulibacter IС and IIС and of *Ca.* Accumulibacter IIB among the PAO. Based on the content of the genes of the key metabolic pathways, the genomes of potential PAO belonging to the genera *Amaricoccus, Azonexus, Thauera, Zoogloea, Pinisolibacter*, and *Siculibacillus* were selected. The patterns of physicochemical processes and the microbiome structure associated with granule formation and succession of the microbial communities were revealed.

## 1 Introduction

Phosphorus is an important biogenic element, which plays a key role in the constructive and energy metabolism of all living organisms. Its increased recovery and agricultural application resulted in two major problems: eutrophication of water reservoirs due to phosphorus release with wastewater (Farley, 2012) and the danger of global phosphorus shortage since its mineral resources are limited and not renewable (Cordell and White, 2013). To address these problems, the development and introduction of technologies for removing phosphorus from wastewater to an environmentally safe level and for its repeated application are required (Yuan et al., 2012; Butusov and Jernelöv, 2013; Di Capua et al., 2022). The development and introduction of technologies for phosphate recycling from phosphorus-containing waste will result in the production of phosphate fertilizers, satisfying 15–20% of the worldwide requirements (Yuan et al., 2012; Butusov and Jernelöv, 2013). Among such technologies, those based on biological phosphorus removal and combining biological removal with chemical phosphate precipitation are considered the most efficient and economically attractive ones (Cornel and Schaum, 2009; Hirota et al., 2010; Yuan et al., 2012; Wilfert et al., 2015; Rajesh Banu et al., 2021; Zhang et al., 2022).

Enhanced biological phosphorus removal (EBPR) is carried out by phosphate-accumulating organisms (PAO), which develop in bioreactors under cyclically switched aerobic/anaerobic (anoxic/anaerobic) conditions. Under anaerobic conditions, they consume organic compounds, primarily volatile fatty acids (VFAs), and store them as intracellular polymers, polyhydroxyalkanoates (PHAs), with simultaneous degradation of intracellular polyphosphates and orthophosphate release from the cells. Under aerobic conditions, PAO grow, consume orthophosphate, and synthesize intracellular polyphosphates using the energy derived from the decomposition of the intracellular carbon and energy sources accumulated under anaerobic conditions. *Candidatus* Accumulibacter (phylum *Pseudomonadota*) and members of the genus *Tetrasphaera* (phylum *Actinomycetota*) are considered to be the main agents of EBPR (Hesselmann et al., 1999; Stokholm-Bjerregaard et al., 2017; Fernando et al., 2019). Other PAO, however, probably exist as well and play a significant role in EBPR (Stokholm-Bjerregaard et al., 2017; Dorofeev et al., 2020; Petriglieri et al., 2021; Diaz et al., 2022).

Although PAO have been used for phosphorus removal at large-scale waste treatment plants for half a century, their taxonomic composition, interaction with other microorganisms of the activated sludge consortium, and physiological properties are largely unknown since the major PAO, including *Ca.* Accumulibacter, have not been isolated in pure cultures. Some success in the understanding of PAO metabolism was achieved due to the investigation of the metagenomes of PAO communities (Martin et al., 2006; Petriglieri et al., 2022). PHA synthesis from VFA (e.g., acetate) in the anaerobic phase and its accumulation begin with VFA transport into the cell, which is either passive, involving porin proteins, or active, by acetate permease encoded by the *actP* gene. Further conversion of acetate to acetyl-CoA may occur via either the high-affinity pathway involving acetyl-CoA synthetase coded by the *acs* gene, or via the low-affinity one, by acetate kinase and phosphate acetyltransferase, coded by the genes *ackA* and *pta*, respectively. The subsequent conversion of acetyl-CoA to PHA requires the products of three genes: *phaA* (β-ketothiolase), *phaB* (acetoacetyl-CoA reductase), and *phaC* (PHA synthase; Oyserman et al., 2016). During the aerobic stage, the accumulated PHA is degraded by poly(3-hydroxybutyrate) depolymerase, coded by the *phaZ* gene (Oyserman et al., 2016). Transport systems also play an important role in phosphorus metabolism. PAO possess a low-affinity phosphate transporter pit and a high-affinity phosphate transport system pst (*pstABCS*). The *ppk1* gene-encoding polyphosphate kinase is the most important gene directly related to PAO energy metabolism and required for polyphosphate synthesis in the aerobic phase. Another important gene is *ppk2* (AMP-polyphosphate phosphotransferase), with its product involved in polyphosphate decomposition during the anaerobic stage. Apart from polyphosphate accumulation, glycogen is also stored in the cells. Three key genes of this pathway may be named: *glgA*, *glgB*, and *glgC*; their products are involved in glycogen formation from α-D-glucose-1P. The decomposition of accumulated glycogen is carried out by glycogen phosphorylase (*glgP*).

Technologies of enhanced biological wastewater treatment using aerobic granulated activated sludge became popular recently (Hamza et al., 2022). They have significant advantages compared to the technologies using the conventional flocculated activated sludge: The facilities occupy less area and require lower capital and exploitation costs (De Kreuk et al., 2005; Nancharaiah and Reddy, 2018; Zheng et al., 2020). The mechanisms of granule formation are insufficiently studied. The process of granule formation, their size, and their biochemical properties are known to depend on a number of factors, including reactor configuration and operating regime (hydrodynamic processes, temperature, medium composition, duration of the settling and starvation stages, etc.), and on the dominant species (Weissbrodt et al., 2013; Hamza et al., 2022; Zheng et al., 2022). The efficiency of EBPR was reported to be associated with the granule geometry, decreasing with their increasing size (Wu et al., 2010; Li et al., 2018; Quoc et al., 2021). Significant differences between the flocculated and granulated sludges were revealed at the proteome level (Barr et al., 2016). A number of studies reported that the differences in the microbial composition of the granules affected their sedimentation characteristics (Winkler et al., 2011). Selection of the granules with higher PAO abundance has been suggested as an approach to increased phosphorus removal efficiency (Winkler et al., 2011; Bassin et al., 2012; Henriet et al., 2016). PAO-enriched granules have high density and ash content; they are heavier and precipitate in the reactor better. Removal of excessive biomass from the upper sludge layers makes it therefore possible to achieve higher PAO abundance and to increase phosphorus removal efficiency to 100%.

To control the formation of the granules with desired properties, formation patterns of the aggregated microbial community should be known at all stages of its development, from flocs to mature aggregates. The investigation of these processes will promote the development of strategies for optimal bioreactor operation upon transition to the EBPR technologies with granulated sludge and will increase the efficiency of biological phosphorus removal by highly enriched PAO cultures. Few long-term studies of aggregate formation from activated sludge in the EBPR technologies have been described in the literature (Wu et al., 2010; Yun et al., 2019).

The goal of the study was to investigate the patterns of long-time functioning of a phosphate-accumulating community in an SBR reactor during biological phosphorus removal. Our attention was focused at the investigation of different stages of the formation of granular structures from the flocs of activated sludge without granule selection and at succession patterns of microbial communities and the major PAO.

## 2 Materials and methods

### 2.1 Bioreactor operation

Cyclic cultivation was carried out in a 2-L laboratory reactor (SBR) with a thermostat jacket. The reactor was equipped with the system of aeration and regulated nitrogen supply (Eltochpribor, Russia). Supply and removal of the medium were performed by peristaltic pumps Masterflex L/S (Cole Parmer, United States). Gas flows and peristaltic pumps were regulated automatically using a LOGО universal logical module (Siemens, Germany). Agitation was achieved by a magnetic stirrer (200 rpm) C-MAG MS7 (IKA, Germany). Anaerobic conditions were established by pumping nitrogen (0.3 L min^−1^) into the bioreactor. The temperature was maintained at 18°C and pH at 7.5–8.2.

Every 6-h cycle of operation (SBR cycle) included five phases: (1) medium injection under anaerobic conditions, 30 min; (2) anaerobic phase, 2 h 25 min; (3) aerobic phase, 2 h 30 min; (4) settling phase, 30 min; and (5) supernatant removal, 5 min. In each SBR cycle, 0.8 L of the medium was exchanged, with the hydraulic retention time (HRT) maintained at 15 h. A part of the biomass was removed together with the liquid, and the sludge retention time (SRT) was maintained at 17.5 days.

The inoculum used was flocculated activated sludge from Moscow waste treatment plants operating according to the UCT technology.

### 2.2 Medium for cultivation of the PAO community

The PAO-enriched microbial community was obtained using a synthetic medium with acetate as a carbon source and the Р/С ratio of 0.08 (Kong et al., 2002; Majed and Gu, 2020). The medium contained the following (g L^−1^ tap water): CH_3_COONa·3H_2_O, 0.670; (NH_4_)_2_SO_4_, 0.139; КH_2_PO_4_, 0.109; yeast extract, 0.009; MgSO_4_·7H_2_O, 0.150; and trace elements solution, 1 mL. The trace element solution contained the following (g L^−1^): Na EDTA, 10; FeCl_3_·6H_2_O, 1.5; H_3_BO_3_, 0.15; CuSO_4_·5H_2_O, 0.03; MnCl_2_·4H_2_O, 0.12; Na_2_MoO_4_·2H_2_O, 0.06; ZnSO_4_·7H_2_O, 0.12; KI, 0.18; and CoCl·6H_2_O, 0.15. Thiourea was added to the final concentration of 2.5 mg L^−1^ to suppress nitrification. HCl (0.5 M) was added for pH adjustment (7.5 mL L^−1^).

### 2.3 Analytical techniques

The concentration of dissolved oxygen was measured with an Oxi 197 meter (WTW, Germany). The pH of the medium was measured using an Ekspert-001 pH meter-ion meter (Ekoniks-Ekspert, Russia). Acetate was determined on a Staier HPLC chromatograph (Akvilon, Russia). For this purpose, the sample was centrifuged, and the supernatant was acidified with 5 M H_2_SO_4_ to pH 2. Separation was carried out on an Aminex HPX-87H column (BioRad, United States) in the isocratic mode with 25 mM H_2_SO_4_ as the eluent at the rate of 0.6 mL min^−1^. The signal was registered by a UV detector at 210 nm. The total biomass amount (Total Suspended Solids, TSS), the amount of volatile suspended solids (VSSs), and the concentration of phosphate ions (P-PO_4_^3−^) and phosphorus in the biomass were determined in accordance with the standard methods (APHA, 2017). The balance of phosphorus content in the biomass (Р%) was calculated as follows:


$$P\%=100^{*}\left(P_{\mathrm{in}}-P_{\mathrm{out}}\right)*SRT/\left({TSS}^{*}HRT\right)$$

where *P*_in_ and *P*_out_ are phosphate concentrations in the inflowing and removed media, respectively, SRT is the biomass retention time (days), and HRT is hydraulic retention time (days).

### 2.4 Microscopic techniques

Microscopy was carried out after the separation of the two aggregate morphotypes. The aggregates were separated manually under a dissection microscope (Mikromed МС-5-ZOOM-LED, Russia). Cell morphology was investigated under an Olympus CX41 phase contrast microscope (Japan).

Electron microscopy of total cell preparations and X-ray microanalysis of the cells were carried out using a JEM-1400 microscope (JEOL, Japan) equipped with an X-ray microanalyzer (Oxford Instruments, Great Britain), at accelerating voltage of 80 keV and sample inclination of 15°. The spectra were analyzed using the AZtec software package (Oxford Instruments, Great Britain). The same program was used for elemental mapping of the samples. The samples for X-ray microanalysis were prepared using copper grids with carbon-coated formvar films. Native cell preparations were applied to the grids, dried, and used for analysis. PAO in the microbial community were determined as the cells containing intracellular granules consisting of phosphorus compounds, as was indicated by the results of X-ray microanalysis. The cells without inclusions and the background phosphorus content on the formvar film were used as the controls. Total preparations were examined under a JSM-IT200 scanning electron microscope. The preparations were prepared as follows: The samples were applied to coverslips, fixed in increasing concentrations of ethanol (30%, 5 min; 50%, 5 min; 70%, 5 min; 96%, 10 min), and dried for 24 h. After gold spraying, the samples were examined at 0.02 keV.

### 2.5 Molecular techniques

DNA was isolated using the DNeasy PowerSoil Pro kit (QIAGEN). PCR amplification of 16S rRNA gene fragments comprising the V3–V4 variable regions was carried out using the universal prokaryotic primers 341F (5′-CCTAYGGGDBGCWSCAG-3′) and 806R (5′-GGACTACNVGGGTHTCTAAT-3′; Frey et al., 2016). The PCR fragments were bar-coded using the Nextera XT Index Kit v.2 (Illumina, San Diego, CA, United States) and purified using Agencourt AMPure beads (Beckman Coulter, Brea, CA, United States). The concentrations of PCR products were calculated using the Qubit dsDNA HS Assay Kit (Invitrogen, Carlsbad, CA, United States). All PCR fragments were then mixed and sequenced on Illumina MiSeq (2 × 300 nt from both ends). Paired overlapping 16S reads were merged using FLASH v1.2.11 into longer reads (Magoč and Salzberg, 2011). Low-quality reads were filtered, high-quality reads were clustered into OTUs at 97% identity threshold using usearch v11, and chimera sequences were removed during the clustering step by the usearch algorithm. All initial reads (including low-quality ones) were mapped to OTU representative sequences at a minimum 97% global identity to calculate OTU size for each sample, and singleton OTUs were removed. Taxonomy for the OTUs was predicted with vsearch v2.14.1 SINTAX algorithm and Silva v138.1 16S database.

Metagenomic reads were *de novo* assembled using metaSPAdes v3.15.4. Assembled contigs were clustered into MAGs using three different binning programs: MaxBin v2.2.7, MetaBAT v2.15, and concoct v1.1.0. The optimized binning scheme was defined by DAS Tool v1.1.4 from the results of the three binning programs. The completeness and contamination of MAGs were assessed using CheckM v1.1.3, and GTDB-Tk toolkit v2.0.0 was used to assign taxonomic classifications to MAGs. KEGG annotation of MAGs was done using the KAAS annotation server.

A total of 14,920,753 paired reads (8982293306) for type I aggregates (day 250) and 12,142,670 paired reads (7309887340) of the bases for the aggregate metagenome (day 400) were obtained.

Hierarchical clustering based on the weighted UniFrac distance matrix was calculated using Usearch v11.

## 3 Results

### 3.1 Bioreactor operation

**Figure 1:**
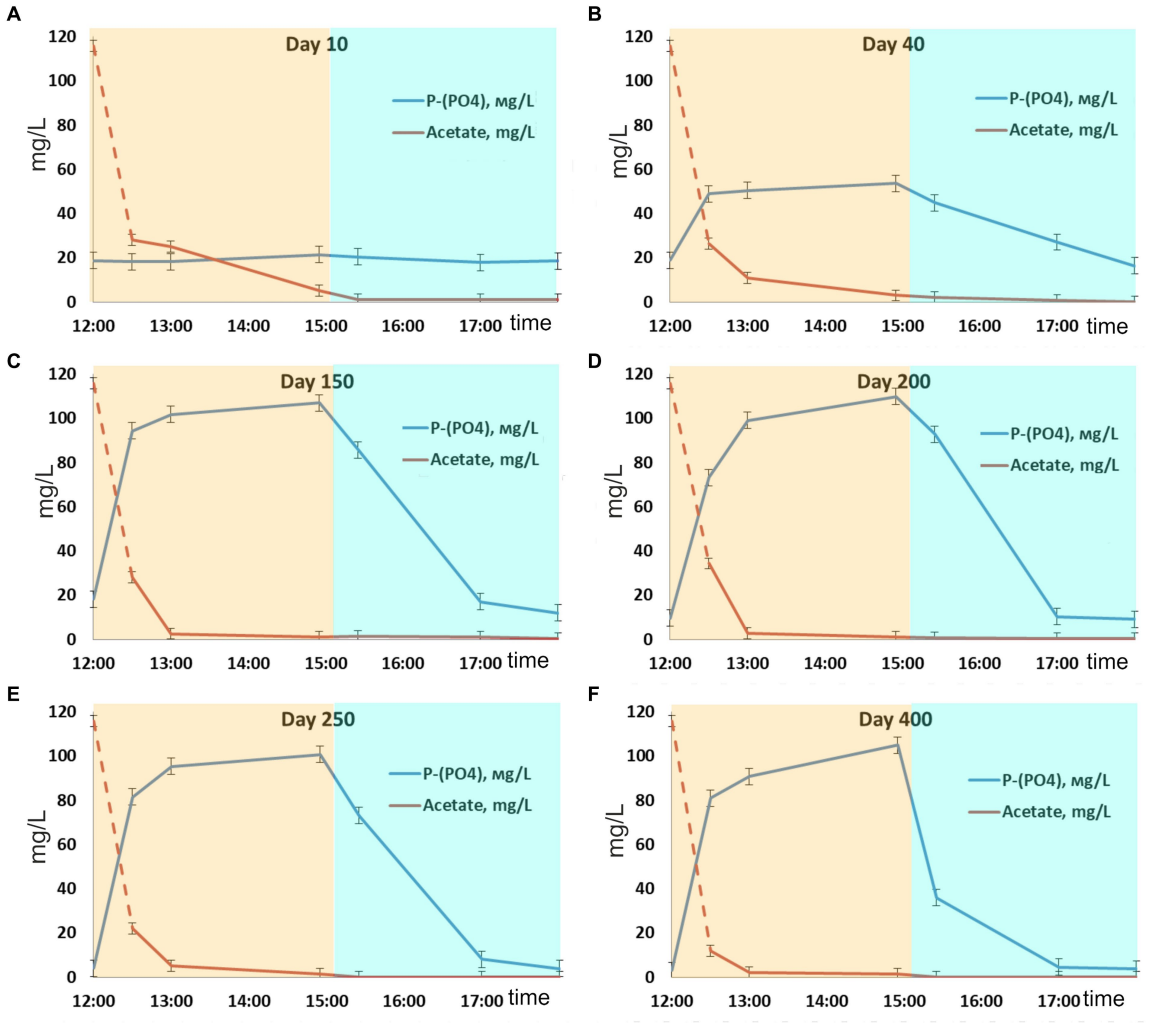
Dynamics of phosphate and acetate concentrations during one SBR cycle: days **(A)** 10, **(B)** 40, **(C)** 150, **(D)** 200, **(E)** 250, and **(F)** 400.

After inoculation with activated sludge, complete consumption of acetate has been observed during the anaerobic phases throughout the experiment. No phosphorus consumption or its release into the medium was observed during the first 10 days of operation, while acetate was consumed (Figure 1), which indicated the activity of heterotrophic members of the community not involved in phosphate accumulation. By day 40, the community was observed to adapt to the SBR cultivation conditions, which resulted in PAO development, as was indicated by cyclic variations of phosphate concentration in the medium. During the anaerobic phase, the concentration of phosphates increased (release), while during the aerobic phase it decreased (consumption; Figure 1). Phosphate concentrations at the end of the anaerobic and aerobic phases were 53.6 and 17.6 P-PO_4_ L^−1^, respectively, while the P-released/acetate uptake ratio was 0.28 P-mol/C-mol (Table 1). During this period, phosphate accumulation efficiency was low (29.6%), with acetate only partially utilized by PAO. After 40 days of the experiment, the biomass concentration (TSS) varied from 3.5 to 4.4 g TSS L^−1^.

**Table 1:** Parameters of SBR operation and the biomass.

Parameter	Days					
	10	40	150	200	250	400
Р_in_, mg L^−1^	25	25	25	25	25	25
P_out_, mg L^−1^	18.7	17.6	15.1	9.4	3.9	3.3
P-removal efficiency, %	25.3	29.6	39.5	62.4	84.3	86.8
Р_max_ anaer, mg L^−1^	21,5	53.6	106.9	109.8	100.7	105.0
P-released/acetate uptake, P-mol/C-mol	<0.1	0.28	0.73	0.77	0.72	0.76
Acetate amount consumed for phosphorus removal, mg Ac/mg P (Ac:P ratio)	47.0	40	29.9	19.0	14.0	13.6
Biomass concentration, g TSS L^−1^ (±10%)	3.1	3.6	4.1	3.5	3.6	4.4
Phosphorus content in the biomass, % of TSS	4.5	8.1	8.5	12.0 (10.6 ± 0.1^*^)	16.0	13.8

^*^Experimental value.

By day 150, phosphorus removal efficiency increased to 39.5%. During the anaerobic phase of the SBR cycle, acetate was completely consumed, similar to the previous period, and phosphate concentration in the medium increased significantly (Figure 1C). By the end of the anaerobic phase, phosphate concentration was as high as 100–110 mg L^−1^, while at the end of the aerobic phase it decreased to 10–15 mg L^−1^. The P-released/acetate uptake ratio reached 0.73 P-mol/C-mol and did not significantly change further.

By day 200, phosphate concentrations at the end of the anaerobic and aerobic phases were 100–110 and 9 mg P-PO_4_ L^−1^, respectively (Figure 1D), while the ratio of released phosphorus to acetate consumed during the anaerobic phase was 0.77 P-mol/C-mol. By day 200, the ash content of the biomass at the end of the aerobic phase of the SBR cycle was 36.0 ± 1.0% от TSS. Phosphorus content in the biomass was 10.6 ± 0.1% of TSS. Calculation of the balance of phosphorus content in the biomass showed values close to the experimental ones (on average 12% TSS). The phosphorus removal efficiency during this period was 62.4%.

During days 250–400 of cultivation, the SBR operation was stable (Figure 1; Table 1). Phosphate concentrations by the end of the anaerobic and aerobic phases were 100–105 and 3–4 mg P-PO_4_ L^−1^, respectively. The phosphorus removal efficiency was 3.4 times higher than at the onset of operation and reached 84–87%. The P-released/acetate uptake ratio varied from 0.72 to 0.76 P-mol/C-mol. The calculated average phosphorus content in the biomass (TSS) was 13.8%. The amount of acetate used for phosphorus removal decreased to 13.6–14.0 mg C mg P^−1^.

### 3.2 Dynamics of the bioreactor microbial community determined by the 16S rRNA gene sequencing

**Figure 2:**
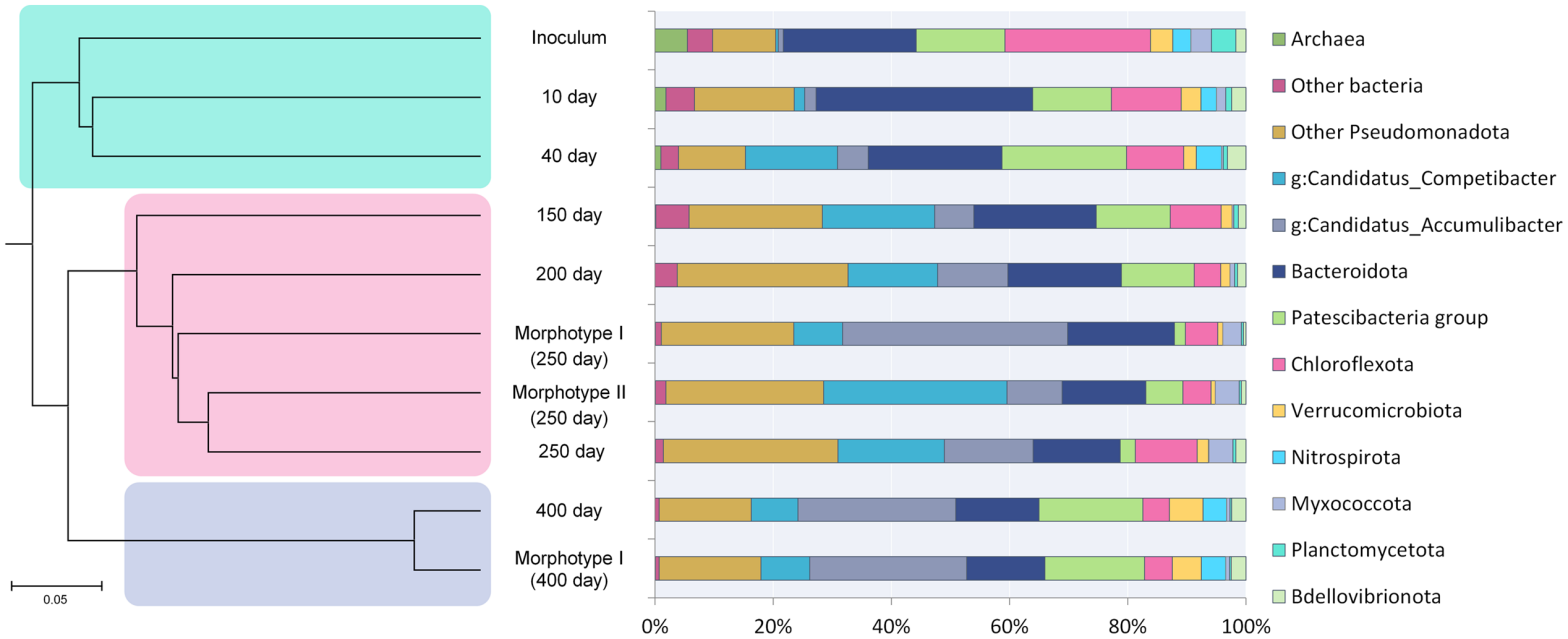
Succession of the microbial community and hierarchical clustering of its members. See the text for explanations.

Changes in the microbial community diversity in the course of the SBR operation are shown in Figure 2. At the onset of the experiment, the highest community diversity was observed, which corresponded to the composition of the inoculum (Table 2). Members of the phylum *Chloroflexota* were the most numerous group, responsible for 19.21% of the total number of the 16S rRNA gene sequences. Representatives of phylum *Bacteroidota* (16.86%), clade *Patescibacteria group* (8.27%), *Pseudomonadota* (9.31%) were shown in smaller numbers. The sequences affiliated with *Chloroflexota* belonged mainly to members of the family *Anaerolineaceae* (18.84%). The phylum *Bacteroidota* was mostly represented by uncultured members of the class *Bacteroidia* (orders *Chitinophagales, Cytophagales*, and *Sphingobacteriales*). Most of the *Patescibacteria group* were represented by members of the *Parcubacteria group* (9.04%). The phylum *Pseudomonadota* was represented primarily by *Gammaproteobacteria* (8.90%), including such typical PAO as *Ca.* Accumulibacter (0.71%) and such GAO as *Ca.* Competibacter (0.29%).

**Table 2:** Alpha diversity of the SBR microbial community at different stages of cultivation.

Sample	chao1	shannon_10
Inoculum	775.1	2.27
10 days	733.1	2.34
40 days	522.6	2.11
150 days	396.3	1.92
200 days	349.8	1.82
400 days	319.1	1.65
Morphotype I aggregates, 250 days	339.3	1.61
Morphotype II aggregates, 250 days	386.7	1.82
Mature aggregates, 400 days	346.2	1.7

The individual OTUs deserving special mention are two OTUs belonging to *Anaerolineaceae* spp. and responsible for 2.51 and 10.29% of the 16S rRNA gene amplicons and *Nitrospira* sp., a nitrogen oxidizer common in treatment plants (2.21%).

By day 10, the relative abundance of *Bacteroidota* was twice that at the onset (28.23%). Representation of *Patescibacteria* remained almost the same (9.2%). Although the share of PAO (*Ca.* Accumulibacter) increased 2.5-fold, it remained low (1.75%), which was in agreement with the data on the chemistry of the process (Figure 1). The relative abundance of *Ca.* Competibacter increased more than 5-fold, to 1.62%. Thus, *Ca.* Accumulibacter and *Ca.* Competibacter were present in almost the same proportions. The number of *Chloroflexota* sequences decreased to 10.35%, with the share of *Anaerolineaceae* decreasing to 7.90%. Members of the phylum *Ca.* Kaiserbacteria became considerably more abundant, from 0.69% (when the reactor was launched) to 4.98%.

By day 40, the relative abundances of members of the phyla *Pseudomonadota* and *Patescibacteria group* increased to 31.19 and 20.50%, respectively. The shares of *Chloroflexota* and *Bacteroidota* decreased to 8.7 and 20.5%, respectively.

Among the most numerous OTUs, two belonged to *Ca.* Competibacter (7.37 and 5.28%), and the total relative abundance of *Ca.* Competibacter was 15.14%. The most numerous OTU belonged to *Ca.* Accumulibacter (3.38%), with the total relative abundance of this group as high as 5.06%.

Other OTUs with relatively high shares in the community belonged to *Nitrospira* (3.12%), *Rhodocyclaceae* sp. (2.01%), and two members of the family *Microscillaceae* (2.8 and 2.63%).

By day 150 of cultivation, the relative abundance of members of the phylum *Pseudomonadota* increased more than 4-fold compared to the inoculum and reached 45.34%. It should be noted that the share of GAO *Ca.* Competibacter, which increased to 18.54%, was responsible for the increased abundance of members of the phylum *Pseudomonadota*, while the share of PAO *Ca.* Accumulibacter increased insignificantly (to 6.43%). Compared to the previous point (40 days), the proportions of members of the *Patescibacteria group* clade and the phyla *Bacteroidota* and *Chloroflexota* decreased to 9.38, 19.62, and 8.18%, respectively. Among the individual OTUs, increased relative abundance was observed for uncultured *Kapabacteriales* sp. (2.91%) and *Holophagaceae* sp. (2.21%).

During the period from day 150 to day 200, the proportion of *Pseudomonadota* increased further, reaching 52.16% of the whole microbial community. The shares of *Pseudomonadota* groups changed, so that *Ca.* Competibacter constituted 14.54%, i.e., their representation in the community decreased, while, on the contrary, that of *Ca.* Accumulibacter increased almost twice, reaching 11.37%. At this stage of operation, the most represented organisms in the activated sludge community of the reactor were *Ca.* Competibacter and *Ca.* Accumulibacter, which coincided with the stabilization of phosphate consumption. The second most represented group (11.43%) belonged to the phylum *Patescibacteria* group. Similar to the previous stage, shares of the phyla *Bacteroidota* and *Chloroflexota* continued to decrease, reaching 18.14 and 4.16%, respectively.

By day 250, the share of members of the phylum *Pseudomonadota* increased further (to 59.13%), with the sequences of members of the class *Gammaproteobacteria* constituting 53.19%. At this stage, the relative abundance of *Ca.* Accumulibacter was 14.72%, while that of *Ca.* Competibacter increased to 19.35%. The abundance of members of the phylum *Bacteroidota* continued to decrease (13.90%). The relative abundance of *Chloroflexota* sequences, on the contrary, increased to 9.89%. By day 250, the share of members of the *Patescibacteria* group decreased sharply (to 1.70%).

By day 400, the total share of the phylum *Pseudomonadota* in the community did not change considerably (48.76%). The proportion of *Ca.* Accumulibacter reached its highest value of 26.28%, while that of *Ca.* Competibacter was 7.80%. It should be noted that the share of members of the phylum *Bacteroidota* decreased to 13.58%, while that of the Patescibacteria group increased to 16.30%. The increased abundance of the phylum *Nitrospirota*, represented solely by members of the genus *Nitrospira* (3.84%), which oxidize nitrite to nitrate, was an unusual finding.

Hierarchical clustering adaptation of the inoculated community was carried out (Figure 2), and three revealed stages of community development coincided with phosphate removal activity. The first stage (days 0–40, green shading in Figure 2) involved the adaptation of the inoculum to selective conditions of the laboratory bioreactor. During the second stage (days 150–250, pink shading in Figure 2), a stable PAO community was formed, with two types of granule-like aggregates; changes in the community composition corresponded to the dynamics of phosphorus removal from the medium. The third stage (day 400, blue shading in Figure 2) was characterized by the redistribution of members of the phylum *Pseudomonadota* and the elimination of type II morphotype aggregates, which had no effect on the phosphorus removal efficiency.

### 3.3 Formation of two types of granule-like aggregates

Apart from the changes in the species diversity of the microbial community during the experiment, the spatial organization (morphology) of activated sludge changed as well. The reactor was inoculated with structurally homogeneous flocculated activated sludge. In the course of cultivation (stage 2, days 150–250), two morphologically distinct types of granule-like aggregates (morphotypes I and II) were formed.

**Figure 3:**
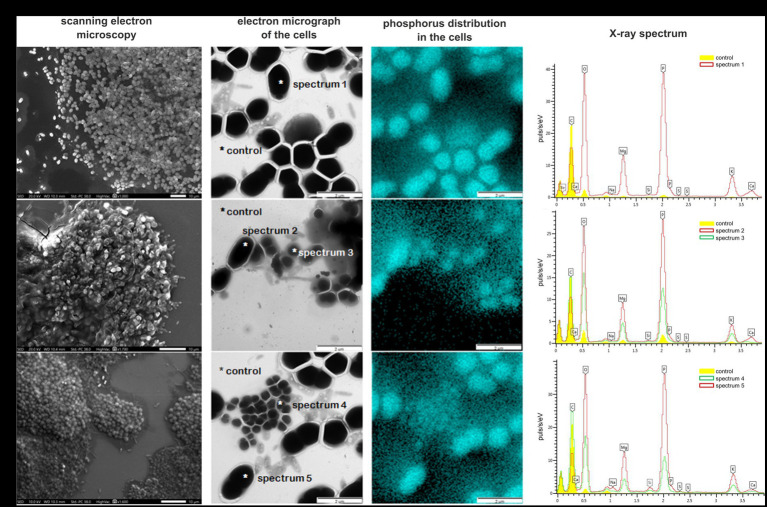
Bacterial diversity in the bioreactor microbial community. Pointwise analysis of the elemental composition of bacterial cells in the phosphate-accumulating community determined by mapping with X-ray microanalysis. Location of phosphorus is marked by a blue color. X-ray spectra of the control (background phosphorus distribution) and spectra 1–5 are concentrations of the elements in the bacterial cells: **(A)** morphotype I (day 250); **(B)** morphotype II (day 250); and **(C)** morphotype I (day 400).

The aggregates of morphotype I were homogeneous flat rounded structures, white-colored, with even edges, varying in size from 0.1 to 0.7 mm (Figure 3A). The aggregates of morphotype II were irregular structures, gray-brown in color, with uneven edges, 0.1–0.5 mm in size (Figure 3B). By day 250, the ratio between the numbers of type I and type II aggregates was 3:1. Scanning electron microscopy revealed that morphologically uniform ovoid bacteria, which predominated in morphotype I granules, were located almost in one plane. In general, morphotype I aggregates resembled bacterial colonies. PAO were identified within the aggregates by the investigation of the elemental composition of the cells using X-ray microanalysis. The community of morphotype I aggregates was relatively homogeneous and consisted of oval cells 1.0 × 1.7 μm in size (Figure 3A). X-ray mapping revealed that each oval cell contained a single inclusion, which occupied almost all its volume (Figure 3A). Pointwise analysis of the elemental composition of these inclusions revealed high levels of phosphorus, potassium, and magnesium (Figure 3A, spectrum 1).

Aggregates of morphotype II had a heterogeneous, three-dimensional structure (Figure 3B). The cells were immersed in a mucous polymer matrix and contained morphologically diverse organisms: cocci and rods of different sizes, as well as filamentous bacteria. Oval cells morphologically resembling those predominant in morphotype I granules were also present (Figure 3B). X-ray mapping of the aggregates (Figure 3B) revealed the presence of phosphorus-containing inclusions in the cells of two different morphologies: large oval and rod-shaped ones. Large oval cells were identical to those predominant in morphotype I aggregates and contained a single large inclusion. Rod-shaped cells were smaller (0.7 × 1.2 μm) and contained two small spherical inclusions. Analysis of the elemental composition of inclusions of both cell types showed that their inclusions contained more phosphorus compared to the control. Phosphorus content in the cells with the single inclusion was twice higher than in those with two inclusions (Figure 3B, spectra 2 and 3, respectively). Apart from phosphorus, the inclusions contained potassium and magnesium. Thus, several morphological cell types were found to accumulate phosphorus, which supported their identification as PAO.

By day 400 of cultivation, selection of the microbial community occurred, resulting in only one aggregate type, morphologically similar to morphotype I, remained in the reactor (Figure 3C). Microscopy (Figure 3C) confirmed the predominance of large oval cells, 1.0 × 1.7 μm in size, similar to those predominant in morphotype I aggregates. Mapping of the microbial community and analysis of the elemental composition also revealed the presence of a single inclusion with a high content of phosphorus and the presence of potassium and magnesium (Figure 3C, spectra 4 and 5, respectively).

### 3.4 Taxonomic composition of microbial communities in the aggregates of different morphotypes

The results of analysis based on the reads of the 16S rRNA gene fragment amplicons are presented in Figure 2. At the phylum level, the diversity of microbial communities in the aggregates of morphotypes I and II was similar, except for the *Patescibacteria* group. The communities of types I and II aggregates contained approximately equal amount of *Pseudomonadota* (66.08 and 64.83%, respectively), *Bacteroidota* (17.16 and 13.53%), *Chloroflexota* (5.16 and 4.52%), *Myxococcota* (2.80 and 2.96%), and *Verrucomicrobiota* (0.82 and 0.71%). The relative abundance of the clade *Patescibacteria* group in morphotype I and morphotype II aggregates was 1.59 and 5.75%, respectively.

Members of the phylum *Pseudomonadota*, to which both PAO and GAO belong, were represented almost equally in type I and type II aggregates, in those of type I morphotype predominated the PAO typical of waste treatment plants, *Ca.* Accumulibacter (37.81%), while potentially phosphate-accumulating members of the genus *Thiothrix* were also present (5.07%). The typical GAOs, *Ca.* Competibacter, were considerably less abundant than PAO (7.85%). In the morphotype II aggregate, on the contrary, GAO *Ca.* Competibacter predominated (30.26%). PAO were represented by *Ca.* Accumulibacter (9.07%) and potentially phosphate-accumulating *Thiothrix* sp. (3.23%). Morphotype I aggregates exhibited lower microbial diversity compared to morphotype II.

Analysis of the aggregates formed by day 400 of cultivation revealed insignificant differences from the morphotype I aggregates developed by day 150. The relative abundance of members of the phylum *Pseudomonadota*, as well as those of *Bacteroidota* and *Chloroflexota*, was comparable to the values observed previously in the aggregates of morphotypes I and II. The share of members of the phylum *Myxococcota* decreased to 0.59%, while that of *Verrucomicrobiota* increased to 4.70%; an increase was also observed for *Nitrospirota* (up to 4.11%) and *Patescibacteria* group (up to 16.30%). In these aggregates, PAO were represented mainly by *Ca.* Accumulibacter (26.15%); no members of the genus *Thiothrix* were detected. The share of *Ca.* Competibacter was also comparable to the value for morphotype I aggregates (7.89%).

### 3.5 Metagenomic analysis of different aggregate morphotypes

**Figure 4:**
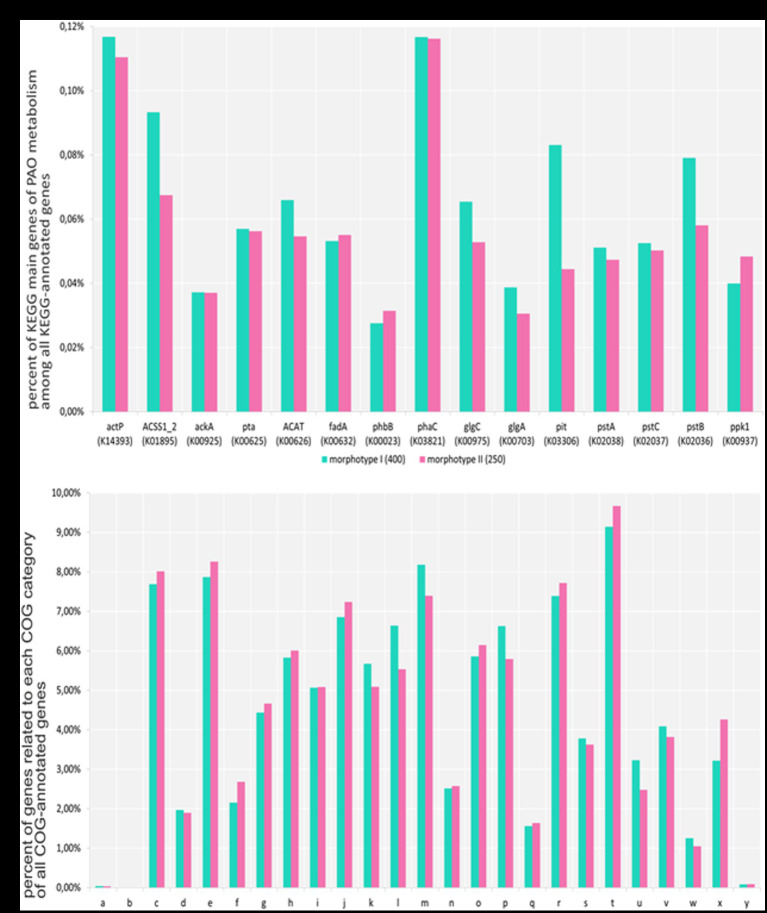
**(A)** Part of all genes that have a KEGG annotation (adjusted for gene multiplicity). Each gene was counted the same number of times as the average coverage of the contig in which the gene located in. 142,738 and 171,060 genes were annotated with KEGG terms for aggregates and PAO samples, respectively. **(B)** Part of all genes that have a COG annotation (adjusted for gene multiplicity). Each gene was counted the same number of times as the average coverage of the contig in which the gene located in. The numbers of genes annotated with COG terms for the aggregates of I and II morphotype samples were 129,529 and 152,663, respectively. A—RNA processing and modification, B—chromatin structure and dynamics, C—energy production and conversion, D—cell cycle control, cell division, chromosome partitioning, E—amino acid transport and metabolism, F—nucleotide transport and metabolism, G—carbohydrate transport and metabolism, H—coenzyme transport and metabolism, I—lipid transport and metabolism, J—translation, ribosomal structure, and biogenesis, K—transcription, L—replication, recombination, and repair, M—cell wall/membrane/envelope biogenesis, N—cell motility, O—posttranslational modification, protein turnover, chaperones, P—inorganic ion transport and metabolism, Q—secondary metabolites biosynthesis, transport, and catabolism, R—general function prediction only, S—function unknown, T—signal transduction mechanisms, U—intracellular trafficking, secretion, and vesicular transport, V—defense mechanisms, W—extracellular structures, X—mobilome: prophages, transposons, and Y—cytoskeleton.

Metagenomic data for the morphotype II aggregates formed by day 250 and mature aggregates similar to morphotype I formed by day 400 were used for functional analysis using the cog database (Figure 4). Our results showed high gene homology in the gene sets of both metagenomes. The metagenome of morphotype II aggregates exhibited higher numbers of transposons and phages, which was an indirect confirmation of the results of amplicons of the 16S rRNA gene fragments indicating higher taxonomic diversity in morphotype II aggregates. A comparison of the pool of the key PAO genes in the aggregates revealed that most of them were present in approximately the same amounts. However, the gene of low-affinity phosphate transporter (pit) was less represented in the metagenome of morphotype II aggregates, which may be an indirect indication of the presence of higher numbers of GAO, compared to morphotype I aggregates. In the metagenome of morphotype I aggregates, the gene coding acetyl-CoA synthetase was less represented, probably indicating the presence of organisms less dependent on acetate consumption (Figure 4A).

For a better understanding of the metabolic patterns of the organisms involved in phosphate accumulation in the samples, genomes were assembled using the metagenome data. For the morphotype I aggregates, eight high-quality draft MAG (Completion >90%, Contamination <5%) and 16 medium-quality draft MAG (Completion ≥50%, Contamination <10%) were assembled and for the morphotype II aggregates 7 and 12, respectively (Bowers et al., 2017). Phylogenetic position of assembled *Ca.* Accumulibacter genomes was specified using the *ppk1* gene sequence. In the morphotype I aggregates, MAG of *Ca.* Accumulibacter belonged to the clades IC and IIC and in the aggregates of another morphotype to IIB. This may be an indication of the predominance of different clades in different aggregates.

Based on the content of the genes of the key metabolic pathways characteristic of PAO and GAO, such as VFA transport (*actP*), VFA conversion to PHA [*actP*, *asc, ackA*, *pta*, *phaA* (*atoB*), *phaB*, and *phaC* (*phbC*)], phosphate transport (*pstA, pstB, pstC,* and *pstS*), and synthesis and degradation of phosphates (*ppk1, ppx*) and glycogen (*glgA, glgB, glgC,* and *glgP*), the genomes of PAO (or potential PAO) and GAO were selected (Ni et al., 2022). The gene of the pit low-affinity phosphate transporter was used as the marker for differentiation between PAO and GAO (McIlroy et al., 2014). The selected genomes belonged to the following phosphate-accumulating bacteria: *Ca.* Accumulibacter IC and IIC, *Azonexus*, *Thauera*, and *Zoogloea* for morphotype I aggregates and *Ca.* Accumulibacter IIB and *Siculibacillus* for morphotype II aggregates.

The genomes lacking the *pit* gene were assigned to potential GAO; they belonged to *Competibacter*, *Amaricoccus*, *Thiothrix*, and *Rhodospirillales* (UXAT02).

Assembled genomes of potential PAO: *Ca.* Accumulibacter IC, *Azonexus*, and *Zoogloea* from morphotype I aggregates and *Siculibacillus* from morphotype II aggregates contained the set of genes required for denitrification (*napA, napB, nirS, norB, norC*, and *norZ*) and may therefore be considered as DPAO. Among the genomes not assigned to PAO, none contained the complete set of denitrification genes. The pathways of dissimilatory nitrate reduction revealed in the MAGs belonging to *Ca.* Accumulibacter, *Azonexus*, *Zoogloea*, *Siculibacillus*, *Bacteroidota* (UBA8401), and *Thiothrix* contained diverse combinations of the genes (*narG, narH, narI, napA, napB, nirB, nirD, nrfA, and nrfH*).

The genes of the sulfur transformation pathways were also found among assembled genomes. The genes of the SOX complex for thiosulfate oxidation (*soxA, soxX, soxY, soxZ, soxB, soxC*, and *soxD*) were found in the MAGs of *Azonexus* and *Zoogloea*. The MAGs of the family *Thiotrichaceae* (*Thiothrix* and *Thiolinea*) contained the genes for assimilatory sulfate reduction (*sat, cysNC, cysN, cysD, cysC, cysH, cysJ*, and *cysI*), while the dissimilatory sulfate reduction pathway (*dsrA, dsrB, aprA, aprB, sat*, and *dsrC*) was found in *Thiothrix*.

Both metagenomic and taxonomic data indicated the predominance of members of the genus *Ca.* Accumulibacter among PAO. More precise determination of the phylogenetic position of assembled *Ca.* Accumulibacter genomes was carried out using the *ppk1* gene sequences. *Ca.* Accumulibacter MAG from type I morphotype aggregates belonged to the clades IC (G, according to GTDB) and IIC (H, according to GTDB), while those from type II morphotype aggregates belonged to IIB. This may indicate the predominance of different clades in different aggregates.

## 4 Discussion

Phosphate dynamics in the SBR cycle indicated that a microbial community highly enriched with PAO, which efficiently removed phosphorus, developed in the reactor by days 150–200. In each SBR cycle, dynamics of acetate concentrations and phosphate release occurred in accordance with the biochemical reactions characteristic of PAO. Under anaerobic conditions, in the presence of the substrate and with no electron acceptors, acetate consumption and phosphorus release occurred (Figure 1). Under aerobic conditions, phosphorus was consumed from the medium. High enrichment with PAO after 150 days of SBR operation was indicated by the high P-released/acetate uptake ratio (0.73–0.77 P-mol/C-mol), which was close to the theoretically calculated values for pH 7.5–8.2 (Smolders et al., 1994; Filipe et al., 2001). This parameter characterizes the ratio of the PAO and GAO biomass: The higher the share of PAO, the higher the P-released/acetate uptake ratio. This ratio in highly enriched PAO cultures grown on acetate is considered to be higher than 0.5 (Brdjanovic et al., 1997; Schuler and Jenkins, 2003a; Bassin et al., 2012; Acevedo et al., 2017). According to this criterion, the community formed in the laboratory bioreactor should be considered a highly enriched PAO community, in which almost all the substrate (acetate) was consumed by PAO.

The high degree of enrichment of the culture with PAO was confirmed by the high ash content of the biomass (36% TSS), which contained a high amount of phosphorus (10.6% TSS or 15.6% VSS), which is characteristic of PAO (Schuler and Jenkins, 2003b; Bassin et al., 2012; Welles et al., 2015). Mañas et al. (2011) showed that at pH 7.8–8.8, high ash content and phosphorus content in the granules were probably associated with chemical precipitation of a portion of phosphates as hydroxyapatite. However, in our experiment, microscopic investigation did not reveal any extracellular mineral particles, either suspended or within cell aggregates. This indicated that phosphorus (as polyphosphates) was mainly located inside the cells.

Changes in the microbial community composition were characterized by an increasing abundance of PAO. By day 400, the share of the 16S rRNA gene fragments of *Ca.* “Accumulibacter phosphatis,” a typical phosphate-accumulating organism, was ~26.28%. At the same time, the share of the 16S rRNA gene fragments of the typical GAO, *Ca.* Competibacter, initially increased up to 18–19% but fell to 7.80% by day 400. Since from day 150 on, most of the substrate delivered into the SBR was consumed in accordance with the PAO metabolism, high GAO abundance indicated that these bacteria had, apart from acetate, an additional source of carbon and energy, which supported their growth. *Сandidatus* Competibacter is known to exhibit some metabolic diversity (McIlroy et al., 2014) and is capable of using acetate, propionate, pyruvate, and some amino acids as carbon and energy sources (Kong et al., 2006). These compounds may be the products of other bacteria within the community, formed via hydrolysis of extracellular biopolymers or autolysis of the dead cells. Representatives of *Bacteroidota* and *Chloroflexota* probably acted as hydrolytics in the PAO community, degrading the high-molecular weight compounds resulting from the metabolism of other bacteria or from the degradation of microbial biomass (Fernández-Gómez et al., 2013; Speirs et al., 2019). This was indicated by the high abundance of the 16S rRNA gene fragments of these microorganisms throughout the experiment. The community contained also relatively many 16S rRNA gene fragments of members of the *Patescibacteria* group. These organisms are known to occur in organic-depleted environments and are characterized by low metabolic potentials and ultra-small cell size (Tian et al., 2020). In our experiments, the *Patescibacteria* group probably played a secondary part in the energy and matter balance, inhabited the zones with oligotrophic conditions, and were responsible for an insignificant part of the total biomass of the consortium.

**Figure 5:**
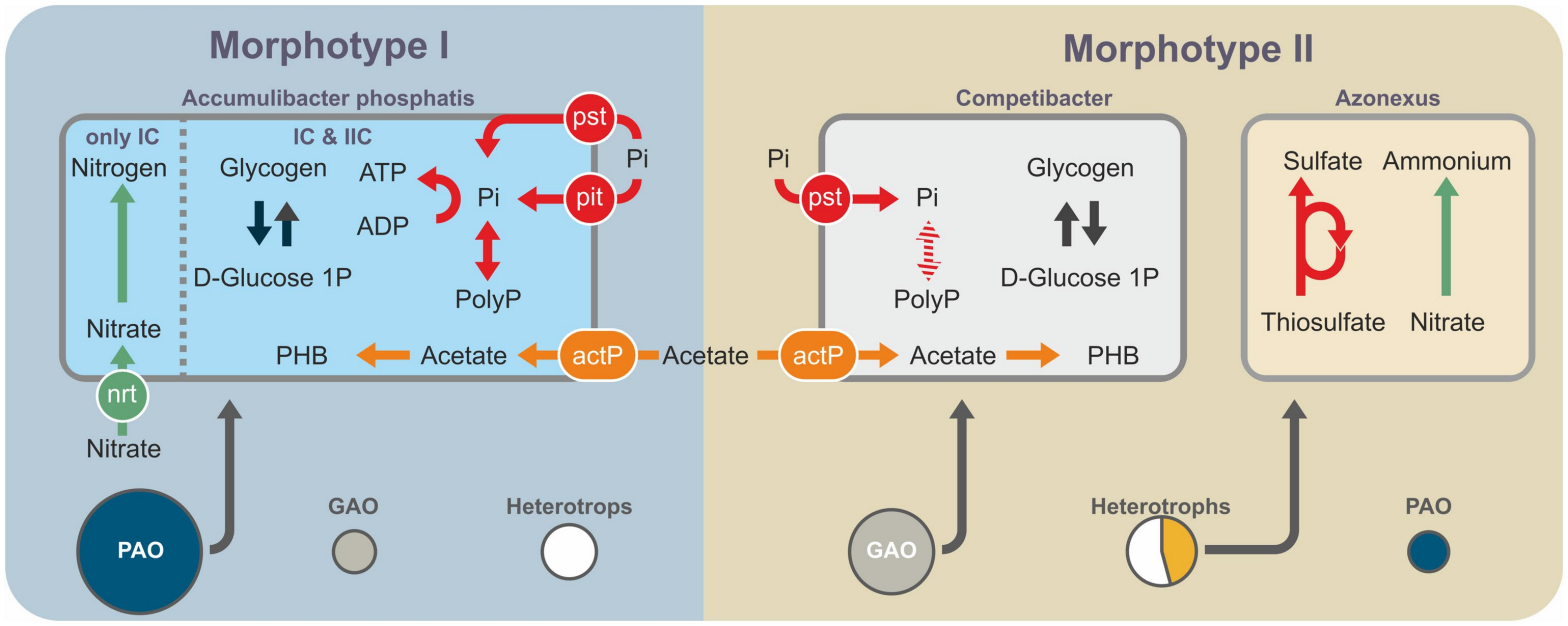
Schematic generalization of the metagenomic data showing the key metabolic pathways and shares of the represented bacterial groups: PAO (violet); GAO (blue); other heterotrophic organisms, except for MT_II-bin.48 (*Azonexus*; gray); and MT_II-bin.48 (*Azonexus*; yellow), since it constitutes a significant part of the metagenome of morphotype I aggregates.

Metagenomic data were used to identify the microbial genomes containing the complete set of genes potentially enabling phosphate accumulation (Figure 5). These included members of the family *Rhodocyclaceae* (*Gammaproteobacteria*): *Azonexus*, *Thauera*, and *Zoogloea*, as well as members of the class *Alphaproteobacteria*: *Siculibacillus*. The results of taxonomic analysis using the 16S rRNA gene amplicons indicated a quantitative predominance of *Ca* Accumulibacter over other potential PAO. Thus, the potential PAO identified based on assembled genomes are involved in the processes of phosphate accumulation as minor components, if at all. The MAGs of potential PAO from mature aggregates belonged to the genera *Azonexus*, *Thauera*, and *Zoogloea* and those from morphotype II granules to *Siculibacillus*.

In activated sludges of waste treatment plants and laboratory bioreactors, phosphate accumulation and denitrification often occur simultaneously and may be carried out by the same organisms, DPAO (Diaz et al., 2022). Among the MAGs assembled in the present study, the genomes of *Ca.* Accumulibacter IC and IIC, *Azonexus*, *Thauera*, *Zoogloea*, and *Siculibacillus* harbor the genes required for both phosphate accumulation and denitrification. These organisms may be therefore considered as potential DPAO.

Our data confirm the modern concepts concerning the absence of the *nar* genes among the denitrification genes of *Ca.* Accumulibacter IC and the presence of the *nar* and *nap* genes in *Ca.* Accumulibacter IIC (Rubio-Rincó et al., 2019; Meng et al., 2023).

*Azonexus*, *Zoogloea*, and *Thaurea* are common in EBPR-active sludges. Analysis of the metagenomic data suggests that *Zoogloea* and *Thaurea* are capable of both denitrification and phosphate accumulation (Mao et al., 2014; Jena et al., 2016; Li et al., 2020; Ren et al., 2021; Wang et al., 2021). At the same time, *Azonexus* is discussed in the literature only as a denitrifying organism (Quan et al., 2006). No confirmation of direct involvement of *Azonexus* in phosphate accumulation has been found.

Common occurrence of *Rhodocyclaceae*: *Ca.* Accumulibacter, *Azonexus*, *Thauera*, and *Zoogloea*, is characteristic of the activated sludges in which granulation occurs due to EPS synthesis and excretion (Lv et al., 2014; Jena et al., 2016; Li et al., 2018 Xia et al., 2018).

*Amaricoccus* occurs in activated sludge and is supposedly a GAO competing with PAO for the substrate. However, it has been reported that *Amaricoccus* does not completely satisfy the requirements of GAO (Falvo et al., 2001). The genome assembled in the present study satisfied the description of GAO, since it contains the genes of VFA consumption and conversion, while not possessing the pit phosphate transporter. The assembled *Amaricoccus* differed from other MAGs in the presence of multiple transport systems for monosaccharides and oligosaccharides.

Another arguable issue is the absence of the *pit* gene in the MAG of *Thiothrix*, which is typical of GAO, although in the literature members of this genus are most often considered as PAO (Rubio-Rincón et al., 2017b).

*Siculibacillus* have been discovered recently and remain insufficiently studied. Our data suggest that these organisms may be potential PAO/DPAO.

### 4.1 Spontaneous formation of granule-like aggregates

A number of external factors are considered to favor granule formation: selection of rapidly settling particles, hydraulic conditions (shear forces), high organic load, addition of carriers and metal ions, and bioaugmentation (Han et al., 2022). In the present study, no harsh conditions for granule formation and selection were applied: The settling time was 30 min (usually 2–10 min), no additives were used, flocculated activated sludge was used for inoculation, and the height-to-diameter (H/D) ratio was 1.0 (column reactors with high H/D values are commonly used). Excessive biomass was removed from the reactor under stirring, together with medium removal, which also limited aggregate selection according to their sedimentation properties. However, the development of the phosphate-accumulating community was accompanied by a gradual transition from flocculated activated sludge (used for inoculation) to granule-like aggregates.

Spontaneous transition of the activated sludge morphology from flocs to granule-like aggregates was observed in SRBs operating in the phosphorus removal mode (Dulekgurgen et al., 2003; Barr et al., 2010; Weissbrodt et al., 2013). Barr et al. (2010) reported aggregate formation after 50–60 days of cultivation under conditions favoring the formation of flocs, rather than granules. Weissbrodt et al. (2013) showed that both the PAO- and GAO-enriched cultures were capable of spontaneous granule formation. Yun et al. (2019) observed the formation of activated sludge granules in the SBRs operating under the modes favoring PAO and denitrifying PAO after 200 and 250 days, respectively. In our experiment, changes in granule morphology were observed somewhat earlier: Granule-like aggregates were detected after 40 days of cultivation. The formation of immature granule-like structures evidently began immediately after the SBR was switched to the mode of enhanced biological phosphorus removal, while the formation of mature granules was completed after several months, depending on the SBR operation mode, medium composition, and the original inoculum (activated sludge).

No clear-cut differences in the gene sets of the key metabolic pathways were observed for identified PAO and GAO in the aggregates of different types. This probably indicates that the formation of granulated sludge is not associated with this pathway or that some minor components of the microbial community, which are not involved in the PAO/GAO processes, play the key role.

The transition from flocs to granules may be considered as a transition to the most efficient form of existence of this microbial community, providing for high stability and complete resource utilization. This is indirectly evidenced by the fact that aggregates (biofilms) are the dominant form of microbial existence in natural environments. This spatial organization significantly increases resistance to unfavorable factors, facilitates the exchange of genetic information, and enhances the role of intercellular communication (quorum sensing; Aliane and Meliani, 2021; Luo et al., 2022). Spatial proximity of microcolonies in biofilms provides better conditions for syntrophic interaction, retaining the extracellular enzymes, preventing leakage of lysed cells’ components, and establishing the concentration gradients of metabolic products, thus increasing the efficiency of their consumption by the microorganisms developing inside the granules (Flemming, 2002; Luo et al., 2022).

The formation of granules and granule-like aggregates is known to depend significantly on the synthesis of extracellular polymer substances (EPSs), which play important roles in biofilms and a key role in the aggregation of activated sludge (Liu et al., 2010; Karygianni et al., 2020; Melo et al., 2022). According to our observations, bacterial cells in the aggregates were embedded in the EPS matrix, which bound the cells together. Although EPSs have been insufficiently studied, extracellular polymers have been shown to carry out important functions in the aerobic–anaerobic cycle of phosphate consumption release. According to some researchers, the extracellular processes involving EPS play a key role in EBPR (Wang J. et al., 2014; Long et al., 2017). Regulation of EPS synthesis in the EBFR microbial communities still remains poorly studied. It is known, however, that at stable temperature and pH, the EPS composition and the efficiency of their synthesis depend considerably on the nature of the organic substrate and the C/N ratio in the medium (Czaczyk and Myszka, 2007; Erkan and Engin, 2019; Yang et al., 2022). Stimulation of EPS synthesis by the activated sludge microbial community was observed upon switching from starvation to abundant substrate (Rahman et al., 2017).

In our opinion, the spontaneous formation of granule-like aggregates in our experiments resulted primarily from the biological patterns of the microbial community functioning under changes from substrate abundance to starvation and from anaerobic to aerobic conditions (feast/famine–anaerobic/aerobic), which is the characteristic of the EBРR technologies. This suggestion is supported by the results of the investigation on the formation of aerobic-activated sludge, which showed that the feast–famine mode together with gravity selection was the major factor responsible for the formation of aerobic aggregates in continuous plug-flow cultures (Kent et al., 2018; Sun et al., 2021). Granule formation may also be facilitated by the development of filamentous bacteria, which are to a significant extent responsible for the structure and sedimentation properties of the activated sludge flocs at WWTP (Wanner et al., 2010; Nierychlo et al., 2019). The aggregates studied in the present study contained filamentous bacteria belonging to *Chloroflexota* and *Thiothrix*, which are common in activated sludge. The structure-forming function of filamentous bacteria was probably pronounced at the early stages of the formation of granule-like aggregates, when these organisms were abundant. The share of *Chloroflexota* in the initial activated sludge was 19.5%, while in mature PAO aggregates it decreased to 4–6%, and *Thiothrix* was absent.

### 4.2 Granule segregation and selection of the PAO aggregates

The development of the microbial community as granule-like aggregates was accompanied by aggregate segregation with the emergence of two morphotypes differing in their morphological characteristics and taxonomic composition. Both types contained PAO and GAO. However, *Ca.* Accumulibacter predominated in morphotype I aggregates, while in morphotype II aggregates *Ca.* Competibacter was dominant. *Candidatus* Accumulibacter in morphotype I aggregates was represented by types IC and IIC and in morphotype II aggregates by type IIB. Microscopic investigation revealed that phosphorus-accumulating cells were present in all aggregates. Morphotype II aggregates were characterized by higher morphological diversity and more complex physical structure; they exhibited similarity to the activated sludge of waste treatment plants. After a long-term coexistence of morphotype I and morphotype II aggregates, the structure of the community changed, and by day 400 it contained only mature morphotype I aggregates.

A comparison of our results with those obtained by Barr et al. (2010) deserves consideration. They reported the first observation of granule segregation into two types: white (compact, smooth, dense, and spherical aggregates) and yellow granules (loose, coarse, and of irregular shape). White granules contained mainly *Ca.* Accumulibacter phosphatis and insignificant amounts of *Ca* Competibacter phosphatis with a small share of other bacteria. *Candidatus* Competibacter phosphatis predominated in yellow granules, while *Ca.* Accumulibacter phosphatis comprised a minority. After the segregation of the granules, the authors used selective washing off to remove more slowly settling aggregates, which resulted in the emergence of hybrid aggregates containing the cells of both PAO and GAO. Importantly, the segregation occurred both under different modes of SBR operation and when different media were used (synthetic medium with VFA or VFA-enriched wastewater). The authors, therefore, concluded that spontaneous segregation was caused by the properties of *Ca.* Accumulibacter phosphatis, rather than with the SBR mode of operation. Our experiments confirmed spontaneous segregation and long-term coexistence of two morphotypes of granule-like aggregates. Subsequently, however, we observed a gradual decrease in the abundance of GAO aggregates, with their complete elimination by day 400 of SBR operation. Unlike the experiments by Barr et al. (2010), the transition to mature aggregates similar to the morphotype I aggregates was spontaneous, without any changes in the SBR operation mode. It should be noted that in our experiments, changes in the community taxonomic composition and selection of the PAO granules continued even after the pseudo-steady-state was established in the SBR, with the stable dynamics of phosphate consumption release after 150–200 days of operation.

The reasons for the long-term coexistence of PAO and GAO aggregates and the coexistence of PAO and GAO within a single granule are of special interest. GAOs are presently considered the main competitors with PAO for the organic substrate under EBPR conditions. Similar to PAO, GAO develop under cyclic aerobic/anaerobic conditions but are incapable of phosphate cycling. Outcompeting of PAO by GAO is, therefore, considered one of the main causes for decreased efficiency of biological phosphorus removal. The result of competition and the ratio between PAO and GAO depend on a number of factors: temperature, type of the organic substrate, organic matter ratio to phosphates, pH (Lopez-Vazquez et al., 2009; McMahon et al., 2010), and the mode of cyclic cultivation (Onnis-Hayden et al., 2020; Izadi et al., 2021; Chen et al., 2022). In our experiments, the physicochemical conditions for the development of PAO were close to optimal: low temperature (18°C), mildly alkaline conditions (pH 7.5–8.2), and the P/COD ratio of 0.08 in the incoming medium. However, the coexistence of the aggregates of both morphotypes was observed for a long time, and subsequently PAO and GAO coexisted in the same type of mature aggregates.

In our opinion, the long-term coexistence of the aggregates of morphotypes I and II and the subsequent replacement of morphotype II reflected the result of competition between microbial communities at the aggregate level. At the first stages (days 0–150), two main types of microbial communities localized in GAO and PAO aggregates developed and coexisted. The most probable materials for their formation are the flocs of activated sludge with different taxonomic compositions. This is indicated by the fact that *Ca.* Accumulibacter cells in PAO aggregates (morphotypes I) belonged to type IC и IIC and those in GAO aggregates of (morphotypes II) to type IIB. Acetate supplied to the SBR is used simultaneously by PAO and GAO. This period is characterized by a gradual increase in the P-released/acetate uptake ratio, which reflected the increased share of PAO aggregates, which subsequently (on days 250–400) complete displacement of GAO aggregates from the SBR. At this stage, almost all supplied acetate was consumed by PAO aggregates, which was indicated by the high value of the P-released/acetate uptake ratio (0.72–0.77 P-mol/C-mol), characteristic of the PAO phenotype. The geometry of PAO aggregates (their flat shape with the higher surface-to-volume ratio) is probably partly responsible for the higher competitiveness of these aggregates, as well as the physiological characteristics of PAO.

**Figure 6:**
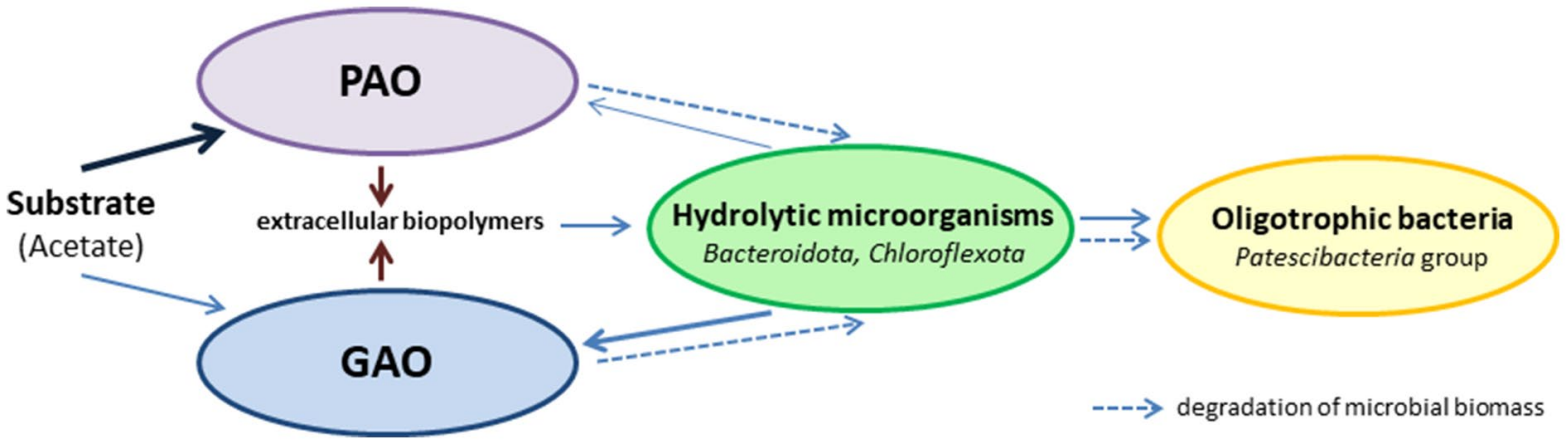
Simplified microbial trophic chain in morphotype I aggregates.

By the end of the SBR operation, mature aggregates similar to those of morphotype I were present in the culture. Although *Ca.* Accumulibacter was predominant, these aggregates contained ~7.89% *Ca.* Competibacter, and no decrease in the GAO relative abundance was observed during a long period. This was an indication of the stable coexistence of PAO and GAO in the same aggregates. Although these groups are usually considered competitors for the substrate, there is no direct evidence either of this or of the negative role of GAO in phosphorus removal (Nielsen et al., 2019). Moreover, GAO and PAO occur together in the activated sludges of wastewater treatment plants and bioreactors. The interactions between PAO and GAO under EBFR conditions are considerably more complex than simple competition for the substrate. Thus, Rubio-Rincón et al. (2017a) showed that under anaerobic conditions, the syntrophic relationship may exist between PAO and GAO, apart from competition. In our opinion, considering the multicomponent composition of PAO aggregates, long-term selection under EBPR conditions results in the selection of the aggregates with stable trophic relationships between PAO, GAO, hydrolytic microorganisms, and oligotrophic bacteria, which determine the stability of this community (Figure 6).

Most of the incoming substrate in these aggregates is utilized by PAO. Hydrolytic microorganisms consume the dead microbial biomass and provide the substrates for GAO and oligotrophic microorganisms. The extracellular biopolymers (EPS) synthesized by the community play a significant part, providing for the structural integrity of the aggregates and acting as carbon and energy sources for the hydrolytic microorganisms.

## 5 Conclusion

Summarizing our results and the literature data, it may be concluded that the long-term functioning of a phosphate-accumulating community in the mode of enhanced biological phosphorus removal is characterized by spontaneous aggregation and segregation of the granules. It is known that granular-activated sludge has advantages over flocculated. However, specific techniques are usually used to obtain granules: selection of rapidly settling particles, specific hydraulic conditions, high organic load, addition of carriers and metal ions, and bioaugmentation. We have shown that the formation of granule-like aggregates in EBPR is a spontaneous process without the use of these techniques, which can significantly simplify the technological process and reduce operating costs.

The phenomena of spontaneous aggregation and segregation of the granules cannot be explained either by simple physical selection of rapidly settling particles or by some unaccounted factors associated with SBR operation (e.g., failure to maintain strict anaerobic conditions during the period when VFA are present). Repeated observations of spontaneous biomass aggregation and segregation in the SBRs using media of different compositions and inoculated with different activated sludges under laboratory conditions indicate that these are phenomena of a basic nature, a typical stage of the development of phosphate-accumulating microbial communities in the EBPR mode. While the pronounced PAO phenotype of the microbial community was already formed during the first months of its development, the subsequent selection of aggregates facilitates a further increase in phosphate removal efficiency. Further in-depth studies of the physiological properties of PAO and GAO are required for the determination of the role of individual members of the community, the dynamics of their quantitative ratios in the aggregates, and their spatial distribution. Thus, the use of a PAO-enriched inoculum to reduce the time to obtain a community with high phosphorus removal efficiency is promising.

## Data availability statement

The datasets presented in this study can be found in online repositories. The names of the repository/repositories and accession number(s) can be found in the article/Supplementary material.

## Author contributions

AB: Formal analysis. AD: Conceptualization, Formal analysis, Methodology. AK: Formal analysis, Validation, Writing – review & editing. AM: Conceptualization, Methodology, Project administration, Resources, Writing – review & editing. AP: Data curation, Formal analysis, Investigation, Original draft. EG: Data curation, Formal analysis, Investigation, Original draft. NR: Conceptualization, Methodology, Writing – review & editing. NP: Conceptualization, Methodology, Project administration, Supervision, Writing – review & editing. YuB: Formal analysis, Writing – review & editing. YuN: Conceptualization, Formal analysis, Methodology, Validation, Writing – review & editing.
